# Oral health evolution in Brazilian adolescents: comparative analysis of SB Brasil Surveys 2003, 2010 and 2023

**DOI:** 10.1590/1807-3107bor-2025.vol39.048

**Published:** 2025-05-19

**Authors:** Andreia Maria Araújo DRUMMOND, Thiago Caldeira DINIZ, Raquel Conceição FERREIRA, Viviane Elisângela GOMES, Rafaela da Silveira PINTO, Mara VASCONCELOS, Marcus Vinícius Camargo PRATES, Andrea Maria Duarte VARGAS

**Affiliations:** (a) Univesidade Federal de Minas Gerais - UFMG, School of Dentistry, Department of Social and Preventive Dentistry, Belo Horizonte, MG, Brazil.; (b) Ministério da Saúde, Departamento de Atenção Primária à Saúde, Coordenação-Geral de Saúde Bucal, Brasília, DF, Brasil.

**Keywords:** Oral Health, Dental Caries, Adolescents, Epidemiology, Health Inequities

## Abstract

This study aimed to compare the findings of the SB Brasil 2003, 2010, and 2023 surveys and analyze the evolution of caries experience among Brazilian adolescents (aged 15–19 years). A total of 16,832 adolescents were evaluated in 2003, 5,445 in 2010, and 8,054 in 2023. Although the 2010 sample was numerically smaller, all surveys maintained representativeness for Brazil, its regions, and state capitals, following national epidemiological sampling criteria. The DMFT index (Decayed, Missing, and Filled Teeth) and its components were analyzed at the national, regional, and state capital levels, considering socioeconomic and regional disparities. A significant reduction in the mean DMFT index was observed, from 5.51 (2003) to 4.25 (2010) and 3.41 (2023), with more pronounced declines in the Northeast and South regions. The decayed component showed a decrease between 2003 and 2023, while the filled teeth without decay component consistently decreased. Despite these improvements, regional inequalities persist, with the North and Central-West regions reporting higher caries rates and lower access to dental care. These findings highlight notable advancements in adolescent oral health in Brazil, particularly regarding the reduction in caries experience across some regions. However, persistent disparities underscore the need for targeted public health policies to ensure more equitable access to oral healthcare.

## Introduction

Dental caries is one of the most prevalent oral health conditions globally, posing a significant challenge to public health systems and individual quality of life.^1^ In Brazil, epidemiological surveillance in this field has advanced through the SB Brasil 2003, 2010, and 2023 surveys, which provide comprehensive data on the prevalence and distribution of oral diseases, including those affecting adolescents aged 15 to 19 years.^2-4^ The monitoring of indicators such as the DMFT index — which reflects the sum of decayed, missing, and filled teeth — makes it possible to track the evolution of oral health over two decades, highlighting both achievements and persistent weaknesses.^5,6^

Similarly, the oral health of the Brazilian population has been analyzed from broader perspectives that go beyond strictly biological or individual dimensions. It is now recognized that social, economic, cultural, and political factors affect the health-disease process, highlighting on oral health inequalities and the social determinants influencing them. This expanded epidemiological perspective supports more effective public policies, such as the National Oral Health Policy.^7,8^

Accordingly, the complexity of the health-disease-care process requires considering how biological, socioeconomic, and cultural elements impact the prevalence of caries and other oral conditions. It is also necessary to investigate the availability, utilization, and access to healthcare services, aspects that directly influence oral health care — a field particularly sensitive to structural and procedural changes capable of reducing inequalities.^6,9^

Despite advancements in recent decades, dental caries remains the primary oral health issue in Brazil, causing pain and tooth loss across various population groups.^10^ In this context, it is essential to strengthen preventive and health promotion programs in oral health, particularly for the most vulnerable communities and age groups.^11^ It is worth noting that, despite the establishment of the Unified Health System (SUS) and initiatives such as the *Brasil Sorridente* program, launched in 2004, gaps still exist in integrating epidemiological indicators into care models, hindering the monitoring and implementation of effective oral health interventions.^2,6,12,13^

The study of the epidemiological profile of oral health in Brazil is, therefore, a key tool for mapping oral health issues and guiding the formulation of public policies and intersectoral strategies, particularly in a country marked by profound socioeconomic and regional inequalities.^14^ In this context, a decline in the prevalence of dental caries has been observed in certain age groups, partly due to increased access to dental services through SUS. However, regional and socioeconomic inequalities persist, as adolescents from the North and Northeast regions continue to show higher caries rates and limited access to dental care, underscoring the need for targeted policies.^6,15,17^

Beyond its clinical consequences, dental caries can profoundly impact on the quality of life of adolescents, affecting factors such as academic performance, social relationships, and psychological well-being.^17-19^ Factors such as dietary habits, oral hygiene practices, and fluoride availability also play a crucial role in the caries experience, highlighting the importance of preventive and educational programs.^20^

In this context, the comparative analysis of the SB Brasil 2003, 2010, and 2023 surveys represents a unique opportunity to assess temporal trends in the oral health of Brazilian adolescents. Therefore, this study aims to highlight not only the progress achieved over these years but also the gaps that still persist, particularly regarding socioeconomic and regional inequalities. It is hoped that this will contribute to the improvement of public policies that promote equity in access to dental care and reduce oral health inequities.^4,5,6^

## Methods

This study used secondary data from the national oral health surveys SBBrasil conducted in 2003, 2010, and 2023 by the Ministry of Health. Details regarding the sampling design, sample size calculation, examiner calibration, and data collection procedures can be found in the respective official reports.^2-4^ These surveys broadly followed the recommendations of the World Health Organization for epidemiological surveys in oral health.^21^

For the present analysis, data from the surveys conducted in 2003, 2010, and 2023 were used to evaluate Brazil as a whole and its regions (North, Northeast, Southeast, South, and Central-West). For Brazilian state capitals, the available data were from the SB Brasil 2010 and 2023 editions. The target population consisted of adolescents aged 15 to 19 years.

The main variable of interest was caries experience, measured by the DMFT index (the sum of Decayed, Missing, and Filled Teeth) and its individual components (decayed, filled without decay, and missing). Estimates were stratified by Brazil, regions, and state capitals to enable detailed comparative analyses.

The statistical analysis was conducted using Stata® software, version 18, following the recommended guidelines for complex samples.^22^ Sampling weights, primary sampling units (PSUs), and strata were specified according to each survey, ensuring data representativeness. Means and 95% confidence intervals (95%CI) of the DMFT index were estimated for each survey year (2003, 2010, and 2023). To compare means across different surveys, a hypothesis test for the difference between means was applied (p < 0.05). Additionally, the percentage distribution of the DMFT components (decayed, filled without decay, and missing) was evaluated to identify potential changes in treatment patterns over time. The analyses were stratified to identify regional variations and patterns specific to state capitals, providing a comprehensive understanding of oral health trends among Brazilian adolescents during the study period.

All SBBrasil surveys were approved by research ethics committees and adhered to the guidelines of Resolution No. 466/2012 of the Brazilian National Health Council, ensuring the usage of the Informed Consent Form to participants or their legal guardians, and were ethically conducted in accordance with the Declaration of Helsinki.

## Results

A total of 16,832 adolescents were evaluated in 2003, 5,445 in 2010, and 8,054 in 2023. Although the 2010 sample was numerically smaller, representativeness for Brazil, regions, and state capitals was maintained across all surveys, following the sampling criteria established for nationwide epidemiological studies.^2-4^

**Figure 1:**
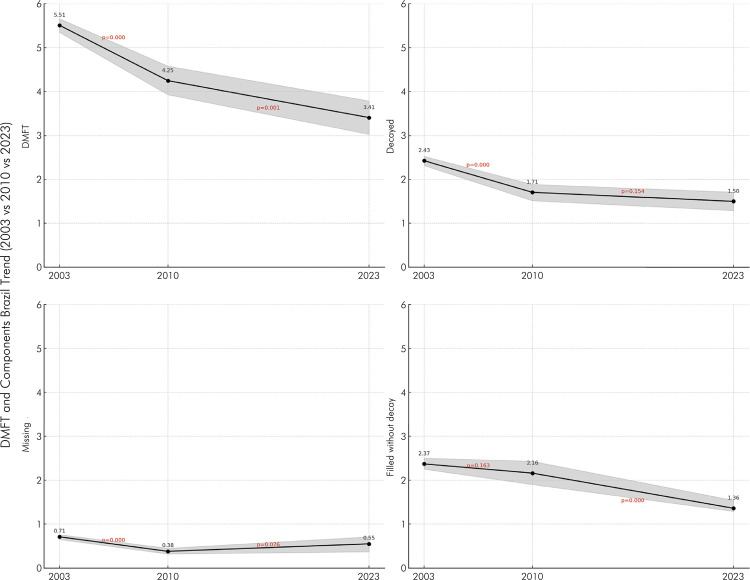
Trend of DMFT and its components (decayed, missing, and filled without decay) among 15 to 19-year-olds in Brazil across the years 2003, 2010, and 2023 (mean values, 95% confidence intervals, and p-values presented).

A statistically significant reduction (p < 0.05) was observed in the DMFT index (sum of decayed, missing, and filled teeth), with the means decreasing from 5.51 in 2003 to 4.25 in 2010 and 3.41 in 2023. The decayed component showed a significant decrease between 2003 and 2010 but did not maintain this trend in a statistically significant manner in 2023. Conversely, the filled without decay component decreased from 2.16 in 2010 to 1.36 in 2023 (p < 0.05). Meanwhile, the missing teeth component dropped by nearly 50% between 2003 and 2010 (from 0.71 to 0.38) but recorded a slight increase to 0.55 in 2023, which was not statistically significant (Figure 1).

The stratified analysis by regions showed significant reductions (p < 0.05) in the DMFT index over the evaluated period, particularly in the Northeast, Southeast, and South regions. In contrast, the North and Central-West regions did not exhibit statistically significant variations, maintaining indices similar to those initially observed (p > 0.05).

The North region recorded a significant reduction in the number of missing teeth between 2003 and 2023 (p = 0.000). Meanwhile, the Northeast region showed a substantial reduction in the DMFT index, from 5.82 ± 0.08 to 3.59 ± 0.24, along with a significant decrease in all components between 2003 and 2023. The Southeast region exhibited a significant decrease in the mean DMFT index across all evaluated years, including reductions in all components from 2003 to 2023. However, no statistical differences were observed in the decayed and missing teeth components from 2010 to 2023, only in the filled without decay component. Similarly, the South region maintained reductions in the DMFT index and its components during the analyzed years, except for the missing teeth component, which did not show significant changes from 2010 to 2023. Finally, the Central-West region demonstrated a significant decrease in the DMFT index and the decayed teeth component from 2003 to 2023, but no statistical differences in the DMFT index or its components were observed between 2010 and 2023 (Table 1).

**Table 1:** Year/Comparison and statistical data (mean, standard deviation, confidence intervals with lower and upper limits, and p-values) for DMFT, decayed, missing, and filled without decay components among adolescents aged 15 to 19 years in Brazil and regions, based on SBBrasil 2010 and 2023 surveys.

Year/Comparison	DMFT				Decayed				Missing				Filled without decay			
	Mean and standard deviation	95%CI		p-values	Mean and standard deviation	95%CI		p-values	Mean and standard deviation	95%CI		p-values	Mean and standard deviation	95%CI		p-values
		LL	UL			LL	UL			LL	UL			LL	UL	
Brazil																
2003	5,51 ± 0,08	5,35	5,66		2,43 ± 0,05	2,32	2,53		0,71 ± 0,03	0,65	0,76		2,37 ± 0,06	2,25	2,5	
2010	4,25 ± 0,17	3,93	4,58		1,71 ± 0,10	1,52	1,89		0,38 ± 0,03	0,32	0,45		2,16 ± 0,14	1,9	2,43	
2023	3,41 ± 0,19	3,03	3,79		1,50 ± 0,11	1,29	1,71		0,55 ± 0,08	0,37	0,71		1,36 ± 0,09	1,29	1,54	
2010–2003	-1,25 ± 0,18	-1,61	-0,89	**0**	-0,72 ± 0,11	-0,94	-0,5	**0**	-0,32 ± 0,04	-0,41	-0,24	**0**	-0,21 ± 0,15	-0,51	0,09	0,163
2023–2003	-2,10 ± 0,21	-2,51	-1,69	**0**	-0,93 ± 0,12	-1,16	-0,69	**0**	-0,16 ± 0,09	-0,33	0,01	0,073	-1,01 ± 0,11	-1,23	-0,79	**0**
2023–2010	-0,85 ± 0,26	-1,35	-0,34	**0,001**	-0,21 ± 0,15	-0,49	0,08	0,154	0,16 ± 0,09	-0,02	0,34	0,076	-0,80 ± 0,16	-1,12	-0,48	**0**
North Region																
2003	5,81 ± 0,17	5,49	6,14		3,47 ± 0,15	3,17	3,77		1,31 ± 0,08	1,16	1,46		1,03 ± 0,09	0,86	1,21	
2010	5,64 ± 0,23	5,19	6,1		3,33 ± 0,24	2,85	3,8		0,95 ± 0,08	0,79	1,11		1,37 ± 0,13	1,1	1,63	
2023	4,80 ± 0,53	3,77	5,84		2,54 ± 0,36	1,84	3,25		0,76 ± 0,12	0,54	0,99		1,50 ± 0,24	1,03	1,96	
2010–2003	-0,17 ± 0,29	-0,73	0,39	0,556	-0,14 ± 0,28	-0,7	0,41	0,612	-0,36 ± 0,11	-0,58	-0,14	**0,001**	0,34 ± 0,16	0,02	0,65	**0,037**
2023–2003	-1,01 ± 0,55	-2,09	0,07	0,068	-0,93 ± 0,39	-1,7	-0,16	**0,018**	-0,54 ± 0,14	-0,81	-0,27	**0**	0,46 ± 0,25	-0,03	0,96	0,067
2023–2010	-0,84 ± 0,58	-1,97	0,29	0,145	-0,78 ± 0,43	-1,63	0,07	0,071	-0,18 ± 0,14	-0,46	0,09	0,189	0,13 ± 0,27	-0,41	0,66	0,640
Northeast Region																
2003	5,82 ± 0,08	5,65	5,98		3,06 ± 0,08	2,9	3,21		1,03 ± 0,04	0,96	1,11		1,73 ± 0,07	1,59	1,86	
2010	4,53 ± 0,21	4,12	4,94		2,34 ± 0,16	2,03	2,64		0,54 ± 0,07	0,41	0,67		1,65 ± 0,09	1,47	1,83	
2023	3,59 ± 0,24	3,11	4,06		1,79 ± 0,21	1,39	2,2		0,72 ± 0,13	0,46	0,97		1,08 ± 0,15	0,79	1,36	
2010–2003	-1,29 ± 0,23	-1,73	-0,85	**0**	-0,72 ± 0,17	-1,06	-0,38	**0**	-0,49 ± 0,08	-0,64	-0,34	**0**	-0,08 ± 0,12	-0,3	0,15	0,517
2023–2003	-2,23 ± 0,26	-2,73	-1,73	**0**	-1,26 ± 0,22	-1,69	-0,83	**0**	-0,31 ± 0,14	-0,58	-0,05	**0,020**	-0,65 ± 0,16	-0,97	-0,33	**0,001**
2023–2010	-0,94 ± 0,32	-1,57	-0,32	**0,003**	-0,54 ± 0,26	-1,05	-0,03	**0,036**	0,18 ± 0,15	-0,11	0,47	0,227	-0,58 ± 0,17	-0,92	-0,24	**0**
Southeast Region																
2003	5,10 ± 0,15	4,8	5,39		1,83 ± 0,07	1,69	1,98		0,41 ± 0,03	0,36	0,472		2,85 ± 0,14	2,58	3,13	
2010	3,83 ± 0,26	3,32	4,34		1,24 ± 0,13	0,99	1,48		0,31 ± 0,05	0,21	0,413		2,28 ± 0,23	1,84	2,73	
2023	2,85 ± 0,33	2,2	3,49		1,13 ± 0,15	0,83	1,43		0,45 ± 0,19	0,09	0,82		1,26 ± 0,14	0,99	1,53	
2010–2003	-1,27 ± 0,30	-1,86	-0,68	**0**	-0,60 ± 0,15	-0,88	-0,31	**0**	-0,10 ± 0,06	-0,22	0,01	0,084	-0,57 ± 0,27	-1,09	-0,04	**0,034**
2023–2003	-2,25 ± 0,36	-2,96	-1,54	**0**	-0,70 ± 0,17	-1,03	-0,37	**0**	0,04 ± 0,19	-0,33	0,41	0,83	-1,59 ± 0,20	-1,98	-1,2	**0**
2023– 2010	-0,98 ± 0,42	-1,81	-0,16	**0,02**	-0,11 ± 0,20	-0,5	0,28	0,592	0,14 ± 0,19	-0,24	0,53	0,454	-1,02 ± 0,27	-1,54	-0,5	**0**
South Region																
2003	5,13 ± 0,12	4,89	5,37		1,84 ± 0,06	1,73	1,95		0,46 ± 0,02	0,43	0,49		2,83 ± 0,07	2,68	2,97	
2010	4,01 ± 0,33	3,37	4,65		1,42 ± 0,18	1,07	1,78		0,21 ± 0,04	0,13	0,28		2,38 ± 0,21	1,96	2,8	
2023	2,35 ± 0,33	1,7	3		0,72 ± 0,10	0,52	0,92		0,27 ± 0,10	0,08	0,46		1,36 ± 0,23	0,9	1,82	
2010–2003	-1,12 ± 0,35	-1,8	-0,43	**0,001**	-0,70 ± 0,21	-1,11	-0,29	**0,029**	-0,26 ± 0,04	-0,34	-0,18	**0**	-0,45 ± 0,23	-0,89	0	**0,05**
2023–2003	-2,78 ± 0,35	-3,48	-2,08	**0**	-1,12 ± 0,12	-1,35	-0,88	**0**	-0,19 ± 0,10	-0,39	0	0,053	-1,47 ± 0,24	-1,95	-0,99	**0**
2023–2010	-1,66 ± 0,47	-2,58	-0,75	**0**	-0,41 ± 0,19	-0,79	-0,04	**0,001**	0,06 ± 0,11	-0,14	0,27	0,551	-1,02 ± 0,32	-1,65	-0,4	**0,001**
Central-West Region																
2003	6,80 ± 0,19	6,42	7,18		2,87 ± 0,18	2,51	3,23		0,65 ± 0,06	0,53	0,77		3,28 ± 0,12	3,05	3,52	
2010	5,94 ± 0,35	5,25	6,64		2,96 ± 0,47	2,03	3,9		0,38 ± 0,04	0,29	0,46		2,60 ± 0,22	2,17	3,04	
2023	5,27 ± 0,64	4	6,53		2,05 ± 0,29	1,48	2,62		0,53 ± 0,09	0,36	0,71		2,68 ± 0,38	1,94	3,43	
2010–2003	-0,86 ± 0,40	-1,65	-0,07	**0,033**	0,10 ± 0,51	-0,9	1,09	0,851	-0,27 ± 0,07	-0,42	-0,13	**0**	-0,68 ± 0,25	-1,17	-0,19	**0,007**
2023–2003	-1,54 ± 0,67	-2,86	-0,21	**0,023**	-0,82 ± 0,34	-1,49	-0,15	**0,017**	-0,12 ± 0,11	-0,33	0,1	0,278	-0,60 ± 0,40	-1,38	0,18	0,13
2023–2010	-0,68 ± 0,73	-2,12	0,77	0,357	-0,91 ± 0,56	-2,01	0,18	0,101	0,16 ± 0,10	-0,04	0,35	0,118	0,08 ± 0,44	-0,78	0,94	0,855

**Figure 2:**
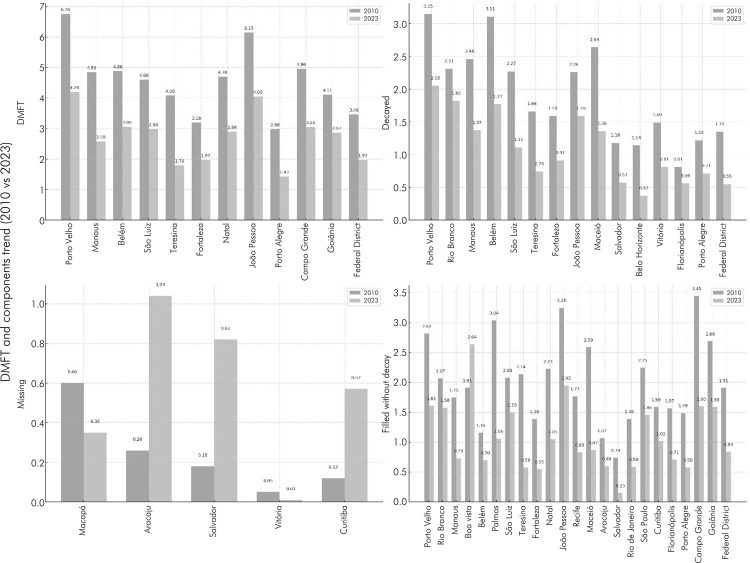
Trend of mean DMFT and its components (decayed, missing, and filled without decay) (p < 0.05) among 15 to 19-year-olds in Brazilian capitals between 2010 and 2023.

A similar situation was observed in the comparison between 2010 and 2023 in Brazilian state capitals, with some registering significant reductions in the DMFT index and its components (p < 0.05), while others showed no statistically significant variations (p > 0.05) (Figure 2; Table 2). The cities of Aracaju, Salvador, and Curitiba experienced a significant increase in the number of missing teeth (from 0.26 to 1.04; 0.18 to 0.82; and 0.12 to 0.57, respectively) (Table 3), while Boa Vista recorded a significant increase from 1.91 to 2.64 in the number of filled teeth without decay (Table 5). Notable examples include the cities of São Luís, Teresina, Maceió, Salvador, Belo Horizonte, and the Federal District, which reported a reduction of 50% or more in the average number of decayed teeth between 2010 and 2023 (Table 4, Figure 2).

**Table 2:** Data on caries experience (DMFT) in Brazilian state capitals: mean, standard deviation, confidence intervals (lower and upper limits), and p-values for the 15 to 19 age group, based on SBBrasil 2010 and 2023 surveys.

Capitals	2010				2023				
	Mean	Standard deviation	95%CI		Mean	Standard deviation	95%CI		p-value
			LL	UL			LL	UL	
Porto Velho	6,76	0,14	6,47	7,05	4,2	0,42	3,34	5,05	**0**
Rio Branco	4,93	0,08	4,77	5,09	4,14	0,51	3,07	5,21	0,142
Manaus	4,85	0,05	4,74	4,96	2,58	0,35	1,86	3,3	**0**
Boa vista	5,68	0,13	5,42	5,94	6,24	0,6	5,01	7,47	0,367
Belém	4,88	0,02	4,84	4,92	3,06	0,45	2,13	3,98	**0**
Macapá	4,02	0,05	3,91	4,12	3,91	0,37	3,16	4,66	0,773
Palmas	5,31	-	-	-	4,46	1,46	1,49	7,43	0,564
São Luiz	4,6	0,13	4,34	4,86	2,98	0,25	2,46	3,5	**0**
Teresina	4,08	0,19	3,7	4,47	1,79	0,26	1,27	2,31	**0**
Fortaleza	3,19	0,07	3,05	3,34	1,97	0,26	1,44	2,5	**0**
Natal	4,7	0,15	4,4	5	2,89	0,28	2,32	3,46	**0,001**
João Pessoa	6,15	0,12	5,91	6,39	4,05	0,57	2,9	5,2	**0**
Recife	3,9	0,22	3,46	4,35	3,21	0,37	2,46	3,96	0,116
Maceió	5,5	0,1	5,29	5,71	4,51	1,29	1,88	7,15	0,451
Aracaju	2,59	-	-	-	3,01	0,42	2,15	3,87	0,322
Salvador	2,09	0,05	1,99	2,19	1,55	0,31	0,92	2,18	0,095
Belo Horizonte	2,33	0,03	2,28	2,39	2,02	0,43	1,16	2,89	0,48
Vitória	2,67	0	2,66	2,68	1,88	0,43	0,98	2,78	0,085
Rio de Janeiro	3,04	-	-	-	2,44	0,67	1,05	3,82	0,382
São Paulo	4,21	0,16	3,9	4,52	3,74	0,44	2,86	4,63	0,322
Curitiba	2,6	0,03	2,53	2,66	2,37	0,27	1,84	2,91	0,408
Florianópolis	2,57	0,11	2,35	2,78	2,13	0,39	1,35	2,91	0,282
Porto Alegre	2,98	-	-	-	1,43	0,3	0,82	2,04	**0**
Campo Grande	4,96	0,03	4,91	5,01	3,05	0,22	2,61	3,5	**0**
Cuiabá	4,31	0,2	3,91	4,71	3,93	0,76	2,39	5,47	0,625
Goiânia	4,11	-	-	-	2,87	0,51	1,82	3,91	**0,022**
Federal District	3,46	-	-	-	1,97	0,27	1,42	2,53	**0**

**Table 3:** Data on missing teeth in Brazilian state capitals: mean, standard deviation, confidence intervals (lower and upper limits), and p-values for the 15 to 19 age group, based on SBBrasil 2010 and 2023 surveys.

Capitals	2010				2023				
	Mean	Standard deviation	95%CI		Mean	Standard deviation	95%CI		p-value
			LL	UL			LI	LS	
Porto Velho	0,79	0,02	0,75	0,82	0,54	0,21	0,11	0,98	0,263
Rio Branco	0,55	0,01	0,52	0,57	0,74	0,28	0,15	1,33	0,508
Manaus	0,64	0,01	0,61	0,67	0,49	0,16	0,16	0,81	0,335
Boa vista	0,75	0,03	0,69	0,8	0,95	0,18	0,57	1,33	0,29
Belém	0,61	0,03	0,55	0,67	0,59	0,2	0,17	1,01	0,922
Macapá	0,6	0,04	0,51	0,69	0,35	0,07	0,2	0,51	**0,008**
Palmas	0,48				1,76	1,42	-1,12	4,64	0,372
São Luiz	0,26	0,02	0,22	0,3	0,37	0,15	0,07	0,68	0,464
Teresina	0,29	0,02	0,25	0,33	0,47	0,22	0,02	0,91	0,425
Fortaleza	0,21	0,02	0,17	0,24	0,51	0,51	0,14	0,89	0,109
Natal	0,71	0,02	0,67	0,75	0,75	0,14	0,18	0,77	0,113
João Pessoa	0,64	0,01	0,62	0,65	0,51	0,17	0,16	0,85	0,449
Recife	0,67	0,04	0,59	0,76	0,81	0,29	0,23	1,4	0,632
Maceió	0,27	0,01	0,25	0,3	2,29	1,66	1,66	5,68	0,235
Aracaju	0,26				1,04	0,24	0,56	1,52	**0,002**
Salvador	0,18	0,01	0,15	0,2	0,82	0,31	0,21	1,44	**0,041**
Belo Horizonte	0,19	0,01	0,18	0,2	0,56	0,37	-0,2	1,31	0,334
Vitória	0,05	0	0,05	0,05	0,01	0,01	-0,01	0,02	**0**
Rio de Janeiro	0,34				0,86	0,57	-0,31	2,04	0,367
São Paulo	0,37	0,02	0,32	0,42	0,53	0,21	0,11	0,95	0,463
Curitiba	0,12	0	0,11	0,12	0,57	0,22	0,12	1,02	**0,049**
Florianópolis	0,18	0,04	0,11	0,26	0,86	0,35	0,16	1,56	0,06
Porto Alegre	0,27				0,14	0,09	-0,04	0,33	0,195
Campo Grande	0,23	0	0,23	0,24	0,18	0,04	0,1	0,27	0,234
Cuiabá	0,28	0,06	0,16	0,4	0,6	0,17	0,26	0,94	0,08
Goiânia	0,23				0,32	0,24	-0,17	0,81	0,714
Federal District	0,2				0,59	0,21	0,16	1,03	0,078

**Table 5:** Data on filled without decay teeth in Brazilian state capitals: mean, standard deviation, confidence intervals (lower and upper limits), and p-values for the 15 to 19 age group, based on SBBrasil 2010 and 2023 surveys.

Capitals	2010				2023				
	Mean	Standard deviation	95%CI		Mean	Standard deviation	95%CI		p-value
			LL	UL			LI	LS	
Porto Velho	2,82	0,11	2,59	3,06	1,61	0,4	0,79	2,42	**0,007**
Rio Branco	2,07	0,02	2,02	2,12	1,58	0,21	1,15	2,02	**0,031**
Manaus	1,75	0,03	1,69	1,81	0,73	0,13	0,46	0,96	**0**
Boa vista	1,91	0,14	1,63	0,19	2,64	0,22	2,19	3,08	**0,009**
Belém	1,16	0,05	1,05	1,27	0,7	0,2	0,29	1,1	**0,032**
Macapá	1,5	0,02	1,46	1,53	1,69	0,42	0,83	2,54	0,654
Palmas	3,04	-	-	-	1,06	0,26	0,52	1,59	**0**
São Luiz	2,08	0,1	1,88	2,28	1,5	0,2	1,08	1,91	**0,015**
Teresina	2,14	0,06	2,01	2,27	0,58	0,13	0,32	0,84	**0**
Fortaleza	1,39	0,04	1,31	1,48	0,55	0,07	0,4	0,69	**0**
Natal	2,23	0,08	2,07	2,4	1,05	0,13	0,79	1,32	**0**
João Pessoa	3,25	0,08	3,08	3,42	1,95	0,4	1,14	2,76	**0,003**
Recife	1,77	0,2	1,36	2,17	0,83	0,09	0,64	1,01	**0**
Maceió	2,59	0,1	2,38	2,79	0,87	0,24	0,37	1,64	**0**
Aracaju	1,07	-	-	-	0,6	0,2	0,2	1	**0,024**
Salvador	0,74	0,04	0,66	0,82	0,15	0,03	0,08	0,22	**0**
Belo Horizonte	1	0,01	0,97	1,03	1,1	0,25	0,6	1,61	0,692
Vitória	1,13	0	1,12	1,13	1,07	0,24	0,57	1,56	0,803
Rio de Janeiro	1,39	-	-	-	0,59	0,14	0,3	0,87	**0**
São Paulo	2,25	0,13	2	2,51	1,46	0,28	0,9	2,03	**0,013**
Curitiba	1,59	0,02	1,55	1,64	1,02	0,16	0,7	1,35	**0,001**
Florianópolis	1,57	0,09	1,39	1,75	0,71	0,13	0,44	0,97	**0**
Porto Alegre	1,49	-	-	-	0,58	0,15	0,27	0,89	**0**
Campo Grande	3,45	0,03	3,38	3,51	1,6	0,17	1,26	1,95	**0**
Cuiabá	2,57	0,21	2,13	3	1,76	0,41	0,93	2,59	**0,091**
Goiânia	2,69	-	-	-	1,59	0,28	1,01	2,16	**0,001**
Federal District	1,91	-	-	-	0,84	0,13	0,57	1,11	**0**

**Table 4:** Data on decayed teeth in Brazilian state capitals: mean, standard deviation, confidence intervals (lower and upper limits), and p-values for the 15 to 19 age group, based on SBBrasil 2010 and 2023 surveys.

Capitals	2010				2023				
	Mean	Standard deviation	95%CI		Mean	Standard deviation	95%CI		p-value
			LL	UL			LI	LS	
Porto Velho	3,15	0,05	3,05	3,25	2,05	0,26	1,52	2,58	**0**
Rio Branco	2,31	0,04	2,23	2,4	1,82	0,23	1,34	2,29	**0,045**
Manaus	2,46	0,01	2,43	2,49	1,37	0,14	1,09	1,65	**0**
Boa vista	3,02	0,09	2,83	3,22	2,66	0,29	2,06	3,25	0,239
Belém	3,11	0,02	3,06	3,16	1,77	0,32	1,12	2,42	**0**
Macapá	1,92	0,02	1,87	1,97	1,87	0,23	1,4	2,34	0,834
Palmas	1,79	-	-	-	1,64	0,56	0,51	2,77	0,787
São Luiz	2,27	0,03	2,2	2,33	1,11	0,17	0,76	1,47	**0**
Teresina	1,66	0,18	1,29	2,03	0,74	0,2	0,33	1,16	**0,002**
Fortaleza	1,59	0,06	1,47	1,71	0,91	0,16	0,58	0,12	**0**
Natal	1,76	0,07	1,62	1,9	1,36	0,26	0,82	1,9	0,155
João Pessoa	2,26	0,04	2,19	2,34	1,59	0,21	1,17	2,02	**0,003**
Recife	1,46	0,09	1,29	1,64	1,57	0,2	1,17	1,97	0,624
Maceió	2,64	0,08	2,47	2,81	1,36	0,3	0,74	1,98	**0**
Aracaju	1,26	-	-	-	1,37	0,2	0,95	1,79	0,588
Salvador	1,18	0,02	1,14	1,21	0,57	0,1	0,37	0,78	**0**
Belo Horizonte	1,14	0,02	1,11	1,18	0,37	0,09	0,19	0,55	**0**
Vitória	1,49	0,01	1,48	1,5	0,81	0,24	0,32	1,3	**0,009**
Rio de Janeiro	1,31	-	-	-	0,99	0,19	0,6	0,14	0,106
São Paulo	1,58	0,12	1,35	1,82	1,75	0,27	1,2	2,3	0,573
Curitiba	0,89	0,04	0,81	0,96	0,78	0,16	0,46	1,1	0,518
Florianópolis	0,81	0,02	0,76	0,86	0,56	0,11	0,35	0,77	**0,027**
Porto Alegre	1,22	-	-	-	0,71	0,17	0,35	1,06	**0,006**
Campo Grande	1,28	0,01	1,26	1,29	1,27	0,13	1,01	1,52	0,943
Cuiabá	1,47	0,21	1,03	1,9	1,57	0,31	0,94	2,19	0,793
Goiânia	1,19	-	-	-	0,96	0,18	0,58	1,34	0,225
Federal District	1,35	-	-	-	0,55	0,14	0,26	0,84	**0**

## Discussion

The presented data highlight significant advances in the oral health of Brazilian adolescents, particularly regarding the reduction in caries experience across various regions of the country. These results align with the implementation of public policies such as the Programa *Brasil Sorridente*, which aimed to expand access to dental services within the Unified Health System (SUS), including secondary care procedures, and prioritized preventive and educational actions^2^. As demonstrated by Moyses et al.,^6^ the expansion of dental services has been associated with a reduction in caries rates among adolescents. The impact of *Brasil Sorridente* is widely recognized as one of the key policies that consolidated the inclusion of oral health services into the Family Health Strategy,^7^ fostering structural and organizational improvements in the oral health system. Furthermore, increased awareness of oral health and the presence of educational campaigns may have contributed to these improvements.^17,18^

Despite overall progress, the persistence of regional and socioeconomic inequalities highlights the need for specific and targeted strategies for regions such as the North and Central-West, which consistently exhibit high dental caries rates.^9,11^ These inequalities reflect historical challenges in Brazil, such as the unequal distribution of infrastructure and healthcare services, and are further exacerbated by disparities among ethnic groups, as demonstrated by Drummond et al.,^16^ who identified significant differences in caries experience between White, African descent, Mixed Race, and East Asian adolescents in the 2010 SBBrasil survey. These findings reinforce the importance of regionalized public policies and intersectoral strategies.^15^

It is crucial to integrate these findings into the broader discussion on the social determinants of health and disease. Factors such as income, education, access to treated water, and basic sanitation policies directly influence the oral health of adolescents.^5,11^ Studies indicate that poverty and social exclusion are directly associated with poorer oral health indicators in Brazil.^24^ Therefore, intersectoral approaches that integrate various public policies, including education, social assistance, and sanitation, are indispensable for promoting sustainable improvements in oral health and reducing inequities.^7,13,14^

The promotion of healthy habits, such as proper brushing and reduced sugar consumption, combined with the expansion of water fluoridation coverage, can have a significant impact on reducing the prevalence of dental caries.^20^ The continuous use of fluoride is recognized as an essential factor in caries prevention, particularly among vulnerable populations.^20^ Previous studies demonstrate that educational programs in schools, combined with the distribution of oral hygiene kits, have proven effective in reducing caries experience among adolescents.^18,19^

In addition to general regional patterns, the analysis of Brazilian state capitals revealed specific variations. The cities of Aracaju, Salvador, and Curitiba showed significant increase in the number of missing teeth, while Boa Vista recorded a significant rise in the number of filled teeth without decay. These differences reflect specific challenges faced by these locations, such as inconsistent access to preventive treatments or failure in maintaining previous interventions.^25^ On the other hand, capitals like São Luís, Teresina, Maceió, Salvador, Belo Horizonte, and the Federal District reported reductions of 50% or more in the average number of decayed teeth between 2010 and 2023, highlighting the success of localized interventions and offering potential models for strategies in other regions with greater challenges.^8^

The analysis of the individual components of the DMFT index revealed distinct dynamics. While the reduction in the decayed teeth component was significant between 2003 and 2010, the absence of a significant trend through 2023 indicates the need to strengthen ongoing preventive measures.^10^ The decrease in filled without decay teeth between 2010 and 2023 suggests improvements in the quality and effectiveness of the dental treatments provided.^18^ However, the non-significant increase in the missing teeth component in 2023 highlights the need for continued monitoring of factors associated with tooth loss, which may be related to socioeconomic factors or the quality of care.^18,25^

The findings of this study corroborate previous research highlighting the effectiveness of oral health programs in reducing the prevalence of dental caries, while also revealing the persistence of regional inequalities^11,25^ The disparities observed in the state capitals suggest that generic interventions may not be sufficient, requiring more tailored approaches.^8^ The results reinforce the importance of the social determinants of health theory, demonstrating how socioeconomic and regional factors directly influence oral health outcomes among adolescents.

## Conclusion

The progress observed in the oral health of Brazilian adolescents over recent decades reflects the positive impact of public policies such as the *Brasil Sorridente* program and the Family Health Strategy. However, the analysis highlights the persistence of regional and socioeconomic inequalities, which limit the full reach of these initiatives in more vulnerable regions, such as the North and Central-West. Differences in access to dental services, as well as the concentration of infrastructure and professionals in more developed areas, continue to pose significant challenges.

The integration of preventive and educational actions, combined with the increase of water fluoridation coverage and the strengthening of primary care, emerges as an effective strategy for reducing caries experience among adolescents, as indicated in previous studies. However, the need for regional and intersectoral strategies remains evident, reinforcing the importance of addressing the social determinants of health to mitigate inequalities and promote more equitable outcomes.

Therefore, the continuity and improvement of public policies based on robust epidemiological data are essential to consolidate the progress achieved. Additionally, the inclusion of programs adapted to local realities and investment in oral health education are crucial to ensuring the sustainability of these advances. Strengthening the collaboration between academia, public administration, and civil society presents itself as a promising strategy to overcome structural barriers and foster equity in the oral health of Brazilian adolescents.
